# An Experimental Trial Exploring the Impact of Continuous Transdermal Alcohol Monitoring upon Alcohol Consumption in a Cohort of Male Students

**DOI:** 10.1371/journal.pone.0067386

**Published:** 2013-06-25

**Authors:** Fergus G. Neville, Damien J. Williams, Christine A. Goodall, Jeffrey S. Murer, Peter D. Donnelly

**Affiliations:** 1 School of Medicine, University of St Andrews, St Andrews, Fife, United Kingdom; 2 Department of Oral Surgery, University of Glasgow Dental School, Glasgow, United Kingdom; 3 School of International Relations, University of St Andrews, St Andrews, Fife, United Kingdom; Yale University, United States of America

## Abstract

**Objective:**

To examine the impact of continuous transdermal alcohol monitoring upon alcohol consumption in male students at a Scottish university.

**Method:**

Using a within-subject mixed-methods design, 60 male university students were randomly allocated into three experimental conditions using AUDIT score stratified sampling. Participants in Conditions A and B were asked not to consume alcohol for a 14-day period, with those in Condition A additionally being required to wear a continuous transdermal alcohol monitoring anklet. Condition C participants wore an anklet and were asked to continue consuming alcohol as normal. Alcohol consumption was measured through alcohol timeline follow-back, and using data collected from the anklets where available. Diaries and focus groups explored participants’ experiences of the trial.

**Results:**

Alcohol consumption during the 14-day trial decreased significantly for participants in Conditions A and B, but not in C. There was no significant relative difference in units of alcohol consumed between Conditions A and B, but significantly fewer participants in Condition A drank alcohol than in Condition B. Possible reasons for this difference identified from the focus groups and diaries included the anklet acting as a reminder of commitment to the study (and the agreement to sobriety), participants feeling under surveillance, and the use of the anklet as a tool to resist social pressure to consume alcohol.

**Conclusions:**

The study provided experience in using continuous transdermal alcohol monitors in an experimental context, and demonstrated ways in which the technology may be supportive in facilitating sobriety. Results from the study have been used to design a research project using continuous transdermal alcohol monitors with ex-offenders who recognise a link between their alcohol consumption and offending behaviour.

## Introduction

Beyond the obvious health concerns, alcohol has a complex relationship with violent offending which poses a broader public health issue. This study is the incipient step of a project to explore the application of continuous transdermal alcohol monitors to reduce alcohol-related violence in Scotland. It is expected that the technology will be of most use for offenders for whom alcohol has been a pervasive factor in their crimes. However, before working with this population, it is necessary to examine in a controlled setting how the continuous monitoring of alcohol consumption is experienced by participants, and how these experiences impact upon behaviour. For the current pilot study participants were male students attending a Scottish university. This group was chosen because they approximately match the target group of offending young males in regards age and gender, culture of excessive alcohol consumption (students are known to drink more heavily than their same-age peers, indulge in ‘binge drinking’ [1], and experience negative consequences from alcohol consumption including injury and assault [2]) whilst allowing easy access, recruitment and evaluation in a controlled context.

### Alcohol as a Public Health Concern

Alcohol excess and misuse contributes to a myriad of adverse health and criminal justice outcomes. The World Health Organization (WHO) estimate that the harmful use of alcohol is associated with 2.5 million deaths annually worldwide through its relationship with disease, accidents and violence [3]. Twelve per cent of these deaths are due to intentional injury (violence or suicide). Furthermore, morbidity figures show that 4.5% of the global burden of disease is attributable to alcohol with 7.8% of that related to violence [3]. Whilst alcohol excess does not inevitably cause violence, it is a common factor in many crimes or incidents of violence and is viewed as a risk factor for violence [4]. Violent offenders in Scotland are predominantly young, male [5], and deprived [6], as are their victims [7]. Alcohol misuse, particularly binge-drinking, tends to be most prevalent for this demographic group, thereby shaping offender profiles [8]. Whilst there is a wider public health and social need to tackle these problems, MacAskill and colleagues highlight the potential benefit that addressing alcohol issues in the offending population may have on recidivism [9].

### Addressing the Alcohol Problem

Problems with alcohol may be addressed in a number of ways including, alcohol brief interventions (ABI) [10], [11], and longer more intensive counselling programmes (e.g. Alcoholics Anonymous) [12], [13]. These initiatives rely on the individual to be motivated to change in order to overcome considerable internal and social barriers to success, and to provide an honest self-report of alcohol consumption. One strategy currently employed in both health and criminal justice sectors to support individuals is alcohol monitoring. For instance, breath alcohol interlock ignition devices have recently gained traction as an effective means of preventing recidivism in convicted drink drivers [14]. Additional options include measurement of alcohol in breath, hair, urine and blood, and by testing blood for biochemical markers. However, each of these methods has its limitations. First, with the exception of the biomarker ethyl glucoronide which offers a detection window of 5 days and will pick up consistent heavy drinking [15], they provide a single recent-point-in-time measurement allowing individuals to manipulate their alcohol intake around points of measurement. Second, blood-alcohol concentration measurements are invasive, and techniques involving blood, hair, urine and biochemical markers usually require laboratory analysis that can be expensive. Third, they are inconvenient as they typically require an individual to present frequently for testing which may consequently reduce compliance.

A newer method of monitoring detects unmetabolised alcohol in perspiration [16]. This technology is increasingly being used in the criminal justice sector, particularly in the US [17]. One such transdermal alcohol monitoring device is SCRAMx (Secure Continuous Remote Alcohol Monitor [see www.alcoholmonitoring.com]; hereafter referred to as an ‘anklet’) which is worn on the ankle and takes a reading every 30 minutes, 24 hours a day over a period of 3 months before the unit needs to be replaced [18]. Information is transmitted for analysis securely via 3G technology, a modem, or direct upload, and drinking episodes can be monitored continuously in real-time. Any attempts to remove or tamper with the anklet are recorded in its output through infrared and temperature sensors.

A recent study found that outputs from the anklet are consistent with the results obtained from breathalyser testing [19]. However, whilst transdermal alcohol monitoring has several advantages, the detection time lags slightly behind that of breath-testing due to the added time required for alcohol to appear in sweat. Moreover, transdermal alcohol monitors do not reliably detect alcohol in perspiration below a blood alcohol level of 20mg/dl [20] meaning that individuals can still drink - albeit at very low levels - so the outcome measure is sobriety rather than abstinence.

There is some evidence to suggest that continuous transdermal alcohol monitoring can successfully reduce recidivism for people convicted of Driving Under the Influence (DUI) of alcohol. Loudenburg and colleagues found that incidents of re-arrest for the crime reduced significantly among those wearing an anklet [21]. An additional artefact of the technology was an apparent reduction in levels of domestic violence among those who wore the anklet [22]. This preliminary research suggests that continuous transdermal alcohol monitoring may be a useful addition to the suite of interventions made available to violent offenders who have a problematic relationship to alcohol.

### The Present Research

In Scotland, those most at-risk of violence perpetration and victimisation are young deprived males who consume excessive quantities of alcohol. In an attempt to prevent this alcohol-related violence, continuous transdermal alcohol monitoring technology has been identified as offering an additional option to assist these young men. To date, the technology has not been systematically analysed outside the US. The primary goals of the current pilot study are to examine whether wearing a transdermal alcohol monitor can support individuals to remain sober, and to explore the ways in which it may be supportive. This is operationalized through systematic manipulation of transdermal alcohol monitoring and sobriety requests. It is hypothesised that for participants who are asked to remain sober, those wearing an anklet will be more successful than those without. The results of this study, and our experience of using the technology, will be used to inform the design and implementation of an intervention with volunteer ex-offenders.

## Methods

### Ethics Statement

All participants provided their written consent to participate in all stages of the study. Ethical approval for the research (including the consent procedure) was granted by the University of St Andrews’ School of Medicine Ethics Committee.

### Participants

Undergraduate and postgraduate students at the University of St Andrews were invited to complete an online suite of questionnaire measures examining alcohol consumption and attitudes towards alcohol, including an AUDIT questionnaire [23] and a 14-day alcohol timeline follow-back [24]. Each alcoholic drink that participants reported having consumed was converted into alcohol units using an online alcohol unit calculator (see www.drinkaware.com). Participants for the current study were recruited from this questionnaire sample by asking male respondents whether they would be willing to take part in a second study if they met inclusion and exclusion criteria. Ten participants who had AUDIT scores ≥20 were excluded since they were deemed at risk of alcohol dependence [23] and thus at risk of withdrawal symptoms if they were allocated to one of the non-drinking experimental conditions. Three participants who had AUDIT scores of 0 were excluded as they were considered non-drinkers. On the advice of the anklet manufacturers, participants with diabetes or skin conditions were also excluded. Of the individuals who met the participation criteria and completed the study, 58.5% were considered Low Risk (AUDIT 0–7), 32.1% as potentially Hazardous (AUDIT 8–15), and 9.4% as potentially Harmful (AUDIT 16–19) drinkers [23]. With regards UK alcohol consumption guidelines, 79.2% of participants consumed more than the recommended limit of 3.5 units on any one day during the 14-day baseline period, and 41.5% consumed more than 21 units during either one of the two weeks.

### Experimental Design and Procedure

A within-subject mixed-methods experimental approach is employed to investigate experiences of alcohol monitoring and analyse patterns of consumption over a 14-day period in March 2012. A mixed-methods approach provides additional insights into questions of not only whether the intervention was successful or not in terms of reducing alcohol consumption, but also how and why such outcomes resulted from the intervention. By including the reflexive accounts of participants through diary studies and focus groups, a greater understanding is obtained regarding the subjective and social experiences of wearing a transdermal alcohol-monitoring device.

Eligible participants with the 60 highest AUDIT scores (*M = *8.35, *SD = *4.75, range = 1–19) were randomly allocated to one of three study conditions through a process of stratified sampling according to AUDIT score. There were no significant differences in age, AUDIT scores or units of alcohol consumed during the baseline 14-day period (as measured using the initial questionnaire) between the three conditions. Participants in two conditions (A and B) were asked not to drink alcohol for 14 days, and participants in Condition A additionally wore an anklet that continuously monitored their alcohol consumption. These two conditions were designed to allow us to compare the impact of wearing an anklet upon alcohol consumption when asked to remain sober. Participants in a third condition (C) wore an anklet for 14 days and were asked to continue consuming alcohol as normal. This condition was designed to provide practical experience of monitoring drinking events, and to examine whether merely wearing an anklet would have an effect on alcohol consumption (see Table 1).

**Table 1:** Experimental conditions and alcohol instructions.

Condition	Anklet	Alcohol instructions
A	Yes	No alcohol
B	No	No alcohol
C	Yes	Continue consuming as normal

### Procedure

Participants in the two anklet conditions (A and C) had their devices fitted in a private room within the School of Medicine onto their leg of choice, and were given the opportunity to return the following day for any necessary adjustments. Anklet condition participants were required to return on days 7 and 14 for data downloads. The anklets were removed on day 14. In total, participants in Conditions A and C visited the School of Medicine four times (five if they needed a re-adjustment) including once for a focus group, whilst participants in Condition B visited just once for a focus group. Anklet data was independently analysed by AMS Technologies (the company who manufacture the anklets) who informed the research team when a participant’s data met their criteria for drinking events. These were incidents in which transdermal alcohol concentration (TAC) was recorded as greater than 20mg/dl, and TAC readings were consistent with a pattern of alcohol consumption and metabolism. If day 7 anklet data from Condition A participants revealed alcohol consumption during the previous week (n = 1), then the relevant participants were contacted by telephone and reminded of the study instruction not to drink alcohol.

All participants who were not lost-to-follow up completed four email-diaries and participated in a focus group. These methods were semi-structured in nature such that they were based around key topics, whilst allowing participants the opportunity to raise and discuss novel relevant issues. The pre-determined discussion topics were i) the experience of wearing an anklet (if applicable), ii) participant contact with alcohol during the 14-day trial, and iii) reasons for success or failure to abstain from alcohol consumption. Diary entries were due on days 2, 6, 13 and 16, and were thus designed to capture experiences of anklet fitting, the two weekends during the trial (the second and third diaries were due on Mondays), and reactions to the end of the study.

Each participant took part in an hour long condition-specific focus group within a week of trial completion. Focus group discussion themes were informed by preliminary analysis of the diary entries, thereby facilitating a reflexive discussion of participant experiences [25], [26]. There were three focus groups for Condition A (n_A1_ = 4; n_ A2_ = 5; n_ A3_ = 4), and two each for Conditions B and C (n_ B1_ = 10; n_B2_ = 11 and n_C1_ = 9; n_C2_ = 11 respectively). Having three Condition A focus groups functioned to reduce participant numbers, thereby ensuring that each individual had enough time to discuss both their experiences of wearing an anklet, and their experience of trying to abstain from alcohol. The difference in the number of focus groups between conditions did not affect the qualitative analysis. Each focus group was led by the first author, who was accompanied by either DJW or CAG who took notes to supplement digital audio recordings [27].

Qualitative data from the diaries and focus groups (fully transcribed using a transcription service) were then analysed using procedures based on Thematic Analysis [28] to disentangle themes as they emerged during the course of the analysis. This process was shaped by dual goals; the first was to accurately represent participant experiences without imposing *a priori* categories upon their responses, whilst the second was to approach the material in terms of specific research topics. The analysis therefore functioned as a compromise between the bottom-up approach of Grounded Theory and the top-down approach of Content Analysis [28]. Systematic readings were first used to familiarise the researchers with the data, before initial codes were independently generated by two of the authors (FGN and CAG) relating to its salient features. The codes were then collated into potential themes, and the data re-checked for instances of these themes. These were next examined to see how they functioned in relation to the data, before further analysis refined the specifics of each theme. Names and definitions were then given to each theme, and an extract chosen to exemplify each one. The Thematic Analysis process was therefore highly iterative such that initial coding was regularly reformulated as a consequence of subsequent analysis.

Upon completion of the trial participants were emailed a link to a second online questionnaire. This was a repeat of the baseline questionnaire, and included a 14-day alcohol timeline follow-back [24] to record their alcohol consumption during the trial. All participants who completed the study were given £50 (approximately $US80) to compensate for their time regardless of their alcohol consumption during the trial (i.e. there was no punishment - withdrawal of any amount of money - for violation of study instructions).

## Results

### Preliminary Analysis

**Figure 1:**
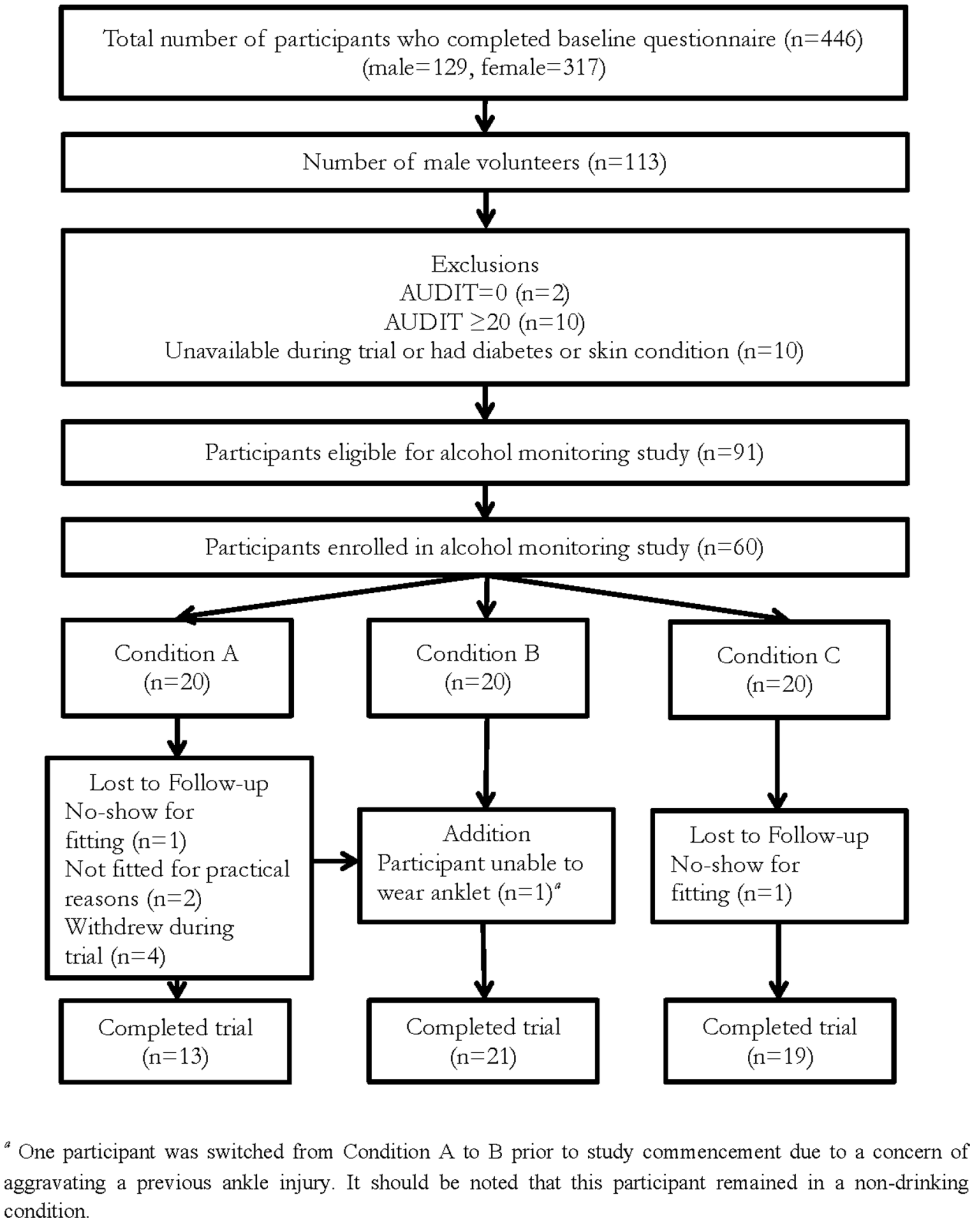
Flow diagram outlining participant recruitment, condition allocation and trial completion.

Of the 60 participants who were allocated to experimental conditions, 53 completed the study and are included in the final analysis (Figure 1). The mean age of participants who completed the study was 21.46 years (SD = 3.51, range = 18–37), with a mean AUDIT score of 7.96 (SD = 4.40, range = 1–17). The AUDIT scores and baseline alcohol units for participants who completed the study, and for those who were lost to follow-up, are reported in Table 2. There were no significant between-condition differences in AUDIT scores or baseline alcohol consumption (as measured using the first questionnaire) for participants who completed the study. In addition, there were no significant main effects of experimental condition or participant loss to follow-up on AUDIT scores or baseline alcohol consumption.

**Table 2:** AUDIT scores and baseline alcohol unit consumption for participants who completed the study, for those who were lost to follow-up [*M*(*SD*)].

	n		AUDIT		Baseline Alcohol Units	
Condition	Completed	Lost to follow-up	Completed	Lost to follow-up	Completed	Lost to follow-up
A	13	7	6.85(5.29)	12.26(5.74)	23.58(24.59)	57.45(43.30)
B	21	0	7.90(4.14)	–	24.85(20.99)	–
C	19	1	8.79(4.02)	4(−)	33.44(18.94)	0(−)

The four participants who began and then withdrew from the study (all in Condition A) had all withdrawn by day 3. Explanations given for withdrawal included feeling uncomfortable, anxious, or dehumanized. None of these participants cited a desire to consume alcohol as their reason for withdrawal. A Fisher’s Exact test (three cells had expected counts <5 preventing a Chi-Square Test) revealed that there was a significant difference in the proportion of participants in each condition who were lost to follow-up, *p<*.01, *V = *.43. For participants in Condition A, the standardised residual (2.50) was greater than the critical value (1.96) indicating that a significantly greater proportion of participants in Condition A were lost to follow-up than expected. There were no significant differences between expected and observed lost to follow-up rates for Conditions B and C.

Within Condition A, participants who were lost to follow-up had a significantly higher mean baseline alcohol consumption (*M = *57.45, *SD = *43.30) than participants who completed the study (*M* = 23.58, *SD = *24.59), *t*(18) = 2.25, *p = *.04, *d = *.96. However, one Condition A participant who was lost to follow-up (the anklet did not fit with his boots which were required for employment) had an outlying alcohol consumption of 132.58 units. When this participant was removed from the analysis, participants who were lost to follow-up (*M = *44.93, *SD = *30.54) no longer had a significantly higher mean baseline alcohol consumption than participants who completed the study (*M* = 23.58, *SD = *24.59), *t*(17) = 1.63, *p = *.12, *d = *.77. Analysis of the anklet data indicated that none of the participants who withdrew met the criteria for alcohol consumption during the period of study in which they had participated.

In the diary entries and post-trial questionnaire, only one Condition A participant reported having consumed alcohol. He recounted six incidents of alcohol consumption, three (50%) of which were confirmed through transdermal alcohol analysis. No other Condition A participants met the transdermal alcohol analysis criteria for a drinking event. Condition C participants self-reported 90 incidents of alcohol consumption. Sixty-eight (75.6%) of these were confirmed as drinking events through transdermal alcohol analysis. Unconfirmed self-report alcohol consumption events were typically incidents in which participants drank a relatively small quantity of alcohol over an extended period, or consumed whilst eating food. As noted in the introduction, SCRAMx transdermal alcohol monitors do not reliably detect alcohol in perspiration below a blood alcohol level of 20mg/dl.

### Quantitative Analysis

Table 3 reports the mean difference in self-report total units of alcohol consumed in each condition during the baseline and trial 14-day periods, and the percentage of participants in each condition who reported having consumed alcohol during the trial.

**Table 3:** Mean self-report total units of alcohol for each condition consumed during baseline and trial 14-day periods [*M*(*SD*)], and percentage of participants who consumed alcohol during the trial.

Condition	Baseline	Trial	Difference (Baseline-Trial)	Consumed Alcohol During Trial (%)
A	23.58(24.59)	3.51(12.67)	20.07*	7.69
B	24.85(20.99)	8.39(13.93)	16.31**	47.62
C	33.44(18.94)	34.30(30.93)	−0.86	94.74

* *p*<.05, **p<.01.

Paired-sample *t*-tests indicated significant decreases in mean self-report alcohol unit consumption during the trial compared to baseline for Condition A; *t*(12) = 2.59, *p = *.02, *d = *1.00, and Condition B; *t*(20) = 3.79, *p*<.01, *d = *0.93, but not for Condition C; *t*(18) = −0.15, *p = *.88, *d* = −0.03.

A one-way ANOVA indicated that there was a significant difference in alcohol unit change (baseline-trial) between conditions, *F*(2,50) = 3.84, *p* = .03 *η^2^* = .13. Tukey post-hoc comparisons of the three conditions revealed that participants in Condition A (M = −20.07, 95% CI [−36.93, −3.21]) reported a significantly larger reduction in units of alcohol consumed during the trial than participants in Condition C (M = −0.86, 95% CI [11.13, 12.86]), *p*<.05. All other comparisons were non-significant at *p*<.05.

A Fisher’s Exact test (one cell had an expected count <5 preventing a Chi-Square test) was used to examine the difference in the proportion of participants in Conditions A and B who consumed alcohol during the trial (counter to study instructions). There was a significant difference in the proportion of participants who consumed alcohol between Condition A (7.69%) and Condition B (47.62%), *p = *.02, *φ* = .42, odds ratio = 10.91, 95% CI [1.19, 99.69]. This between-condition difference was also re-analysed on a conservative ‘intention-to-treat’ basis (i.e. treating all participants who either drank alcohol against study instructions or were lost to follow-up as having ‘relapsed’). Re-doing the analysis in this way identified no significant difference in the proportion of participants who either consumed alcohol or were lost to follow-up between Condition A (40%) and Condition B (40%), χ*^2^* = 0.00, *p = *1.00, *φ* = 0.00, odds ratio = 1.00, 95% CI [0.28, 3.54].

### Qualitative Analysis

Analysis of participant diaries and focus groups suggested several reasons for the smaller proportion of Condition A participants who drank alcohol - contrary to study instructions – compared to those in Condition B. First, several Condition A participants described the physical presence of the anklet as a reminder of their participation in the study and their commitment to abstain from consuming alcohol:

Having the anklet there, it’s always there; you’re reminded of it that you’re taking part in the study. *(Condition A participant - Focus Group A1)*

This was in contrast to the experience of participants in Condition B who recalled incidents in which they had forgotten their participation in the study and drank alcohol:

I attended [a party] and forgot about this study, consuming much of one glass of alcoholic punch before remembering. *(Condition B participant – Diary 2)*

Further, as explained by a participant in Condition A, a perception of being under surveillance discouraged alcohol consumption compared to those in Condition B:

You did feel as though somebody was watching and they’d know if you had a drink. It was always in your mind. I guess if I hadn’t had that [anklet], I could have slipped in a cheeky drink here and there and nobody would know. *(Condition A participant - Focus Group A2)*

This point was developed by a Condition A participant who had his anklet removed for two days during the trial after it was accidentally submerged in water (he claimed not to have consumed alcohol during this period). The participant was therefore in a unique position to compare experiences of the two experimental conditions:

When that anklet is off it’s like no-one’s watching, I can just take a wee dram. The temptation was there a lot more because you think “no-one’s going to find out, no-one cares now, I don’t have the anklet on.” It was a lot harder to not have a drink without the anklet on than with it on. *(Condition A participant - Focus Group A2)*

Finally, a number of participants in Condition A described how they used their anklet as a warrant to resist social pressure from peers for violating norms of alcohol consumption. The symbol [] indicates material omitted from the text for reasons of brevity:

I1: It was a way to explain yourself for not drinking when you were in a situation where drinking was perhaps expected. If you say you’re taking part in a study that’s fair enough, but to have the physical evidence makes it easier. Probably the social pressure again, some people would find it really hard to get away with it. That would be a good reason to say no.

I2: [] having the anklet did make it easier or a more effective way of justifying yourself. *(Condition A participants - Focus Group A1)*

## Discussion

Alcohol poses a significant public health problem in Scotland in terms of its direct impact on health [29], [30] and indirectly through criminal offending [31], [32], particularly violence [33]. Research from the US highlights the potential utility of continuous transdermal alcohol monitoring in aiding the reduction of alcohol consumption in clinical [16] and criminal [17], [21] contexts. This paper has presented data from a pilot study exploring the experience of wearing a continuous transdermal alcohol monitoring anklet and its impact upon alcohol consumption.

Our analysis indicated that participants who were asked not to consume alcohol drank significantly fewer units during the trial compared to baseline, but that there was no significant difference in alcohol consumption for participants who wore an alcohol monitor and were asked to continue drinking as normal. This suggests that simply having one’s alcohol consumption continuously monitored was not enough to change behaviour; some form of instruction recommending a change was necessary to reduce alcohol intake. Among those who were instructed not to drink, there was no significant difference in self-reported units of alcohol consumption between anklet wearers and non-wearers. However, a significantly greater proportion of participants without an anklet violated study instructions and drank alcohol during the trial than those with an anklet, confirming our experimental hypothesis. This difference is likely to be conservative, because whilst transdermal alcohol analysis confirmed that only one anklet-wearer had consumed alcohol, analysis of the non-wearers’ drinking behaviour necessarily relied upon their self-report data.

The results of this pilot study suggest that wearing a continuous alcohol monitor can support individuals who are trying not to consume alcohol. Three possible ways in which the anklets could be effective emerged from analysis of participant diaries and focus groups. The anklets may act as a reminder of the wearer’s commitment to sobriety, the participant may feel under surveillance and thus unable to consume alcohol clandestinely, and some participants reported actively using the anklet as a tool to resist social pressure to drink.

Although the pilot study yielded some interesting findings, these must be treated with caution due to several limitations. First, our sample size was limited due to the cost of monitoring the anklets and compensating participants for their substantial time commitment. However, the trends within the quantitative data were clear and seemed to be validated by analysis of the qualitative work. Second, our sample comprised individuals who would not necessarily be deemed ‘heavy’ drinkers, and thus abstaining from alcohol for 14 days was unlikely to have been a great challenge. This was partly a result of the necessary exclusion of participants with AUDIT scores ≥20 on ethical grounds. As a consequence, few of our participants reported difficulty in remaining sober due to an internal desire for alcohol. Rather, they cited social expectations of alcohol consumption as the biggest threat to their sobriety, and an area in which display of their anklet was useful. Such strategic use of the device may differ in a heavier drinking or dependent population. Nonetheless, all of the participants had AUDIT scores greater than zero, and the mean AUDIT score for participants who completed the study equalled the criterion for hazardous drinking behaviour [22]. Moreover, anecdotally several participants revealed that they had not experienced a 14-day period without consuming alcohol for several years.

A further limitation was the additional time that participants in Condition A spent with the research team compared to those in Condition B, whom we did not meet face-to-face until the post-trial focus groups. It is possible that researcher-participant interactions during the anklet fitting and day 7 data download had an impact upon participant alcohol consumption. For example, participants in Condition A may have felt more guilt at violating the research team’s request for sobriety having met them several times, compared to Condition C participants who only received email contact until the focus groups. Although this additional contact is a potential confounder, it is likely to reflect best practice in future initiatives in which anklet-wearers will receive a package of technical and social support.

The study also suffered a high number of participants who were lost to follow-up. Two participants failed to show up for anklet-fitting (prior to learning their experimental condition), one switched conditions to prevent aggravating an ankle injury, one was unable to wear the anklet with his boots (a requirement for his job) and four withdrew once the trial had begun. None of the participants who withdrew met the criteria for alcohol consumption during the trial (they all gave consent for their anklet data to be analysed up to their point of withdrawal) or cited a desire for alcohol consumption as their reason for withdrawal. However, Condition A participants who had large baseline alcohol consumptions appeared to be the most likely to be lost to follow-up. This suggests that individuals who consume large quantities of alcohol might have difficulty in maintaining and benefiting from continuous alcohol monitoring when asked to remain sober. When the large number of Condition A participants who were lost to follow-up were analysed on an intention-to-treat basis, the between-condition difference for which participants had consumed alcohol became non-significant.

Finally, our findings may not generalise to other populations. Although the current study functioned as a pilot for an intervention for young male offenders, there is some evidence that females are more compliant with sobriety instructions than males [34], [35], and that the anklets show different sensitivity in women [20]. Future work should explore the ways in which people of different ages and genders engage with the technology, and how it might be most effective and supportive for different populations.

### Conclusion

This pilot study examined the impact of continuous transdermal alcohol monitoring upon alcohol consumption in a male student sample from a Scottish University. This was the first time the SCRAMx technology had been systematically analysed outside of the US. Using a mixed-methods experimental design, we demonstrated that significantly more participants who were asked to abstain from alcohol during a 14-day period managed to do so if they wore a continuous transdermal alcohol monitor, compared to those who did not. Qualitative analysis of participant diaries and focus groups explored reasons for this difference. The anklet was found to act as a reminder of participants’ commitment to the study (and the agreement to sobriety), generated a feeling of being under surveillance which contributed to compliance, and was used by some participants as a tool to resist social pressure to consume alcohol. Participants who wore an anklet and were instructed to drink as normal continued to do so. Findings from this study have informed the design of a research project exploring the use of continuous transdermal alcohol monitors to assist ex-offenders reduce recidivism.
